# Correlation Between Gut Microbiota Composition and Serum Interleukin 17 (IL-17) in Mice With Type 2 Diabetes and Experimental Periodontitis

**DOI:** 10.7759/cureus.68005

**Published:** 2024-08-28

**Authors:** Chaolun Mo, Mingkun Huang, Fuhua Yan, Minghui Song, Jiabing Fan, Junmei Zhang

**Affiliations:** 1 Department of Orthodontics, The Affiliated Stomatological Hospital of Guizhou Medical University, Guiyang, CHN; 2 Department of Orthodontics, The Affiliated Stomatological Hospital of Nanjing University Medical School &amp; Nanjing University Institute of Stomatology, Nanjing, CHN

**Keywords:** il-17, proteobacteria, gut microbiota, type 2 diabetes mellitus, experimental periodontitis

## Abstract

Objective

To preliminarily explore the composition characteristics of gut microbiota in mice with type 2 diabetes mellitus (T2DM) and experimental periodontitis, and their correlation with serum IL-17 levels, aiming to provide new insights and evidence for related experimental studies.

Methods

A total of 42 SPF-grade C57BL/6J mice were randomly selected, with 24 used for T2DM modeling. Successfully modeled T2DM mice were divided into the T2DM group (ND group, n=8) and T2DM with experimental periodontitis group (PD group, n=8). Non-T2DM mice were divided into the blank control group (NC group, n=8) and the experimental periodontitis group (NP group, n=8). After modeling, body weight and fasting plasma glucose (FPG) were measured weekly. Each group of mice underwent an oral glucose tolerance test (OGTT) and an insulin tolerance test (ITT). Six weeks after modeling experimental periodontitis, serum IL-17 levels were measured using ELISA, intestinal inflammation was assessed using HE staining, and gut microbiota composition in cecal contents was analyzed by 16S rRNA sequencing to determine its correlation with serum IL-17 levels.

Results

FPG in the PD group was higher than in the ND group, with a statistically significant difference in the 12th week (p<0.05). The glucose tolerance level in the PD group was lower than in the ND group (p<0.01). Compared with the NC group, other groups showed varying degrees of inflammatory cell infiltration in the intestinal mucosa, and serum IL-17 levels were lower in both the ND and PD groups compared to the NC group (p<0.01), with the PD group also lower than the NP group (p<0.01). The Shannon and Pielou-e indices of gut microbiota in the PD group were significantly lower than those in the NP group (p<0.05). In terms of microbiota composition, Firmicutes were increased in both the ND and PD groups compared to the NC and NP groups (p<0.05), while Bacteroidetes were decreased (p<0.05). Proteobacteria were increased in the PD group compared to the ND group (p<0.05). The abundance of Bacteroidetes and the Bacteroidetes/Firmicutes ratio was moderately positively correlated with serum IL-17 levels (p<0.01) and moderately negatively correlated with blood glucose levels (p<0.01); serum IL-17 levels were strongly negatively correlated with blood glucose levels (p<0.01).

Conclusion

Comorbidity of experimental periodontitis and T2DM may exacerbate glucose metabolism impairment in T2DM mice by increasing the abundance of Proteobacteria and intestinal mucosal damage. Serum IL-17 levels may serve as an indicator of gut microbiota dysbiosis in T2DM mice with experimental periodontitis.

## Introduction

Periodontitis is a chronic inflammatory oral disease associated with dysregulation of the plaque biofilm, characterized by a plaque biofilm-induced inflammatory response that leads to the progressive destruction of periodontal supporting tissues [1]. Periodontitis can elevate systemic inflammation levels, influencing the occurrence and progression of systemic diseases [2,3]. Diabetes is a group of metabolic diseases characterized by chronic hyperglycemia resulting from defects in insulin secretion and/or utilization. Type 2 diabetes mellitus (T2DM) is the most prevalent form of diabetes among patients [4]. In patients with poorly controlled T2DM, levels of interleukin 17 (IL-17) and oxidative metabolites in the gingiva are elevated [5]. IL-17 is recognized for its role in immune surveillance at mucosal and barrier surfaces, inducing host defense responses against extracellular bacteria and fungi, and mediating the occurrence and progression of chronic inflammatory diseases such as rheumatoid arthritis, diabetes, and periodontitis [6]. High levels of IL-17 in chronic periodontitis can stimulate the production of pro-inflammatory mediators such as IL-6 and receptor activator of nuclear factor-κB ligand (RANKL), indirectly promoting osteoclastogenesis [7]. In mice with human periodontitis-associated salivary microbiota, *ILC3 cells* significantly inhibit the secretion of IL-17, indicating that oral microbiota dysbiosis can impair spleen immune responses in diabetic mice [8].

There is an interactive influence between periodontitis and diabetes. Diabetes may exacerbate periodontal bone loss by altering the ecological environment of periodontal microorganisms, while oral inflammation and microbial dysbiosis may affect diabetes by inducing gut microbiota imbalance [9]. Periodontitis may influence diabetes by disrupting the ecological balance of the gut microbiota and damaging the intestinal barrier. Numerous clinical studies and animal experiments have confirmed that periodontitis can affect blood glucose control in diabetic patients and animal models, but the mechanisms remain to be further elucidated [10]. Schmidt et al. found that oral microorganisms can enter and colonize the gut, a phenomenon that is common and widespread in healthy individuals [11]. This suggests that even under healthy conditions, oral microbiota may enter and colonize the gut through saliva swallowing. Bao et al.'s study showed that the oral microbiota of periodontitis patients can enter the gut through saliva, leading to changes in the gut microbiota and damage to the intestinal mucosal barrier [12]. Therefore, exploring the characteristics of gut microbiota composition and the correlation with serum IL-17 in T2DM mice with experimental periodontitis is significant for further understanding the "oral-gut-immune axis" mechanism by which periodontitis affects glucose metabolism in T2DM mice.

## Materials and methods

Materials

Experimental Animals

A total of 42 specific pathogen-free male C57BL/6J mice (6-8 weeks old, 20-22 g) were purchased from the Institute of Laboratory Animal Sciences, Chinese Academy of Medical Sciences (license number: SCXK (Jing) 2019-008). The C57BL/6J mice were housed in the Animal Center of Guizhou Medical University under a 12-hour light/12-hour dark cycle at a temperature of 24 ± 1℃, with free access to food and water. The bedding was changed every 2-3 days, and cages and water bottles were cleaned and disinfected. The animal rooms were regularly disinfected with ultraviolet light. All animal experiments involved in this study were conducted in accordance with ethical standards and approved by the Ethics Committee of Laboratory Animal Research at Guizhou Medical University (No. 2201457).

Main Reagents and Instruments

Purified high-fat diet (HFD) (XTHF60, D12492, Xietong Bio); purified standard diet (XTCON50J, D12450J, Xietong Bio); streptozotocin (STZ, Solarbio Technology Co., Ltd., Beijing, China); D (+) -glucose anhydrous (Solarbio Technology Co., Ltd.); ready-to-use anesthetic (1.25% avertin, Aibei Biotechnology Co., Ltd., Guangzhou, China); paraformaldehyde solution (4% PFA, Leigen Biotechnology Co., Ltd., Beijing, China); sodium citrate buffer (0.1 mmol/L, pH=4.5, Solarbio Technology Co., Ltd.); methylene blue (Solarbio Technology Co., Ltd.); recombinant human insulin (Biological Industries, Kibbutz Beit-Haemek, Israel); mouse IL-17 ELISA kit (Cusabio, Houston, USA); low-temperature high-speed centrifuge (Thermo Fisher Scientific, Waltham, USA); microplate reader (Molecular Devices, San Jose, USA); low-speed centrifuge (Hangzhou Mio Instruments, Hangzhou, China); ultrapure water system (Milli-Q, Millipore, Temecula, USA); autoclave (HVE-50, Hirayama, Tokyo, Japan); Illumina high-throughput sequencer (Illumina, San Diego, USA); Roche Accu-Chek glucose meter (Roche, Shanghai, China).

Research methods

Establishment of T2DM Mouse Model

After one week of adaptive feeding, mice with fasting plasma glucose (FPG) ≤ 7.0 mmol/L were numbered and selected for the experiment (n=42). Using a random number table, 24 mice were randomly selected to be fed a high-fat diet (HFD, 60% fat energy), while the remaining 18 mice were fed a standard diet (35% fat energy). After 4 weeks, all mice were fed a normal diet. At the beginning of week 5, all mice were fasted for 12 hours with water allowed. FPG was measured by tail vein blood sampling, and glucose tolerance was assessed by an oral glucose tolerance test (OGTT). At the end of week 5, insulin sensitivity was evaluated by an insulin tolerance test (ITT). OGTT and ITT curves were plotted, and the area under the curve (AUC) was calculated. After successfully inducing insulin resistance in the mice fed with HFD, at the beginning of week 6, the mice were fasted for 12 hours with water allowed. They were then intraperitoneally injected with 30 mg/kg of 2% STZ every other day for a total of three injections. FPG was measured from tail vein blood sampling, with FPG ≥ 11.1 mmol/L on two separate days as the standard for establishing the T2DM mouse model. Mice on a normal diet were intraperitoneally injected with saline at the same time. At this stage, the mice fed with HFD were classified as the HFD group, and the mice fed with the standard diet were classified as the CON group.

Establishment of Experimental Periodontitis Model in Mice

At the beginning of week 7, some T2DM mice and some CON group mice were randomly selected and intraperitoneally injected with 1.25% avertin at 0.2 ml/10 g body weight to create the experimental periodontitis model. After anesthesia took effect, a 5-0 silk suture was tied around the cervix of the right mandibular first molar of the mice and a surgical knot was made. The remaining mice underwent a sham operation: the 5-0 silk suture was removed immediately after ligation. Subsequently, the retention of the silk suture in the experimental periodontitis model mice was observed every 2-3 days, and if detachment was found, it was immediately re-ligated. After 6 weeks of ligation, the mice were sacrificed and the mandibles were isolated and fixed in 4% paraformaldehyde solution for 24 hours. After staining with a 0.5% methylene blue solution for 1-2 minutes, take a photograph using a digital camera. The distance from the cementoenamel junction to the alveolar crest at the mesial and distal sites of the right mandibular first molar was analyzed using Image J software to assess the height of alveolar bone resorption.

Experimental Grouping

The mice on a normal diet (CON group) were randomly divided into two groups: blank control group (NC group, n=8) and experimental periodontitis group (NP group, n=8). The successfully modeled T2DM mice (HFD group) were randomly divided into two groups: T2DM group (ND group, n=8) and T2DM with experimental periodontitis group (PD group, n=8) (Table 1).

**Table 1:** Group and process in the experiment. T2DM: type 2 diabetes mellitus; STZ: streptozotocin; HFD: high fat diet

Group	Experimental Treatment	
	Establishment of T2DM	Establishment of Experimental Periodontitis
Blank Control Group (NC)	No (standard feed + intraperitoneal injection of saline)	No (5-0 silk ligature immediately removed after placement)
Experimental Periodontitis Group (NP)	No (standard feed + intraperitoneal injection of saline)	Yes (5-0 silk ligature, sustained for 6 weeks)
T2DM Group (ND)	Yes (HFD feeding for 4 weeks followed by intraperitoneal injection of 2% STZ)	No (5-0 silk ligature immediately removed after placement)
T2DM with Experimental Periodontitis Group (PD)	Yes (HFD feeding for 4 weeks followed by intraperitoneal injection of 2% STZ)	Yes (5-0 silk ligature, sustained for 6 weeks)

Observation of General Condition, Glucose Metabolism Testing, and Sample Collection in Mice

After the experiment began, the mice's mental state, activity, fur color changes, and bedding dryness were observed every other day. Body weight and FPG were measured weekly. After 6 weeks of establishing the experimental periodontitis model, all groups of mice were fasted for 12 hours with free access to water. FPG was measured, and OGTT and ITT were performed as previously described. Subsequently, each group of mice was anesthetized with 1.25% avertin by intraperitoneal injection, and blood was collected from the heart. After cardiac blood collection, the mice were euthanized by cervical dislocation, and sterile dissection was performed to collect cecal contents in pre-cooled 2 mL sterile cryopreservation tubes, which were immediately snap-frozen in liquid nitrogen and stored at -80°C for future analysis. Part of the small intestine tissue was fixed in 4% paraformaldehyde solution for 24 hours for HE staining, and part was stored at -80°C for later use. Finally, the mandibles were isolated and fixed in a 4% paraformaldehyde solution for future use.

Serum IL-17 Detection

Blood was collected from the hearts of the mice and left at room temperature for over 30 minutes to clot. The clotted blood was then centrifuged at 1000 g for 10 minutes at 4°C, and the supernatant was collected. Serum IL-17 levels were determined using a mouse IL-17 ELISA kit. Samples were added strictly according to the kit instructions, and absorbance at 450 nm was measured using a fully automated microplate reader to create a standard curve and calculate the corresponding IL-17 concentrations.

Histopathological Examination of Small Intestine Tissue

The fixed small intestine tissue of the mice was dehydrated using a graded ethanol series, routinely embedded in paraffin, sectioned, dehydrated, HE stained, and coverslipped for observation of intestinal tissue structure under a light microscope.

Gut Microbiota Diversity Analysis

Total DNA was extracted from the cecal contents of mice and quantified using Nanodrop, with the DNA quality checked by 1.2% agarose gel electrophoresis. Primers were designed based on the conserved regions of the microbial ribosomal RNA target sequence, with sample-specific barcode sequences added. The variable region of the rRNA gene was then amplified by polymerase chain reaction (PCR), using the forward primer ACTCCTACGGGAGGCAGCA and the reverse primer GGACTACHVGGGTWTCTAAT. DNA polymerase was used for PCR amplification, targeting the 16S V3V4 region, with the amplified sequence length limited to 292-480 bp. The PCR-amplified products were quantified using fluorescence and the sequencing library was prepared and purified using Illumina's TruSeq Nano DNA LT Library Prep Kit. High-throughput sequencing was performed on the Illumina NovaSeq platform to sequence the community DNA fragments in a paired-end manner. High-throughput 16S rRNA sequencing of mouse cecal contents was performed, with species richness represented by Chao1 and Observed species indices, species diversity by Shannon index, and species evenness by Pielou's evenness (Pielou-e) index. The raw high-throughput sequencing data were preliminarily screened and subjected to appropriate re-sequencing and supplementary sequencing. Qualified raw sequences were classified into libraries and samples using index and barcode information, with barcode sequences removed. Quality control, denoising, merging, and chimera removal were performed using DADA 2. Alpha diversity levels were assessed based on the distribution of amplicon sequence variants (ASVs) or operational taxonomic units (OTUs) across different samples. Statistical tests were conducted on distance matrices using various unsupervised sorting and clustering methods to measure beta diversity differences, species abundance composition differences, and their significance between different groups. Differential species and marker species between groups were analyzed using linear discriminant analysis (LDA), with dimensionality reduction scores and linear discriminant analysis effect size (LEfSe) calculated.

Statistical Analysis

Experimental data were statistically analyzed using SPSS 26.0 software (IBM Corp., Armonk, USA), and statistical plotting was done using GraphPad Prism 9 software (GraphPad, San Diego, USA). Numerical variables were tested for normality; if all groups met the normality assumption, the data were statistically described using mean ± standard deviation (± SD). Comparisons of means among multiple groups were conducted using repeated measures ANOVA or one-way ANOVA. If normality was not met, the data were described using the median (interquartile range, P_25_-P_75_), and the Kruskal-Wallis H test was used for comparisons among multiple groups. Post-hoc pairwise comparisons were performed using the Tukey method or Bonferroni correction for significance level adjustment. Spearman correlation analysis was used to examine the correlation between bacterial phyla and genera with IL-17 and blood glucose levels. A p-value ≤ 0.05 was considered statistically significant.

## Results

Evaluation of the T2DM Mouse Model

**Figure 1:**
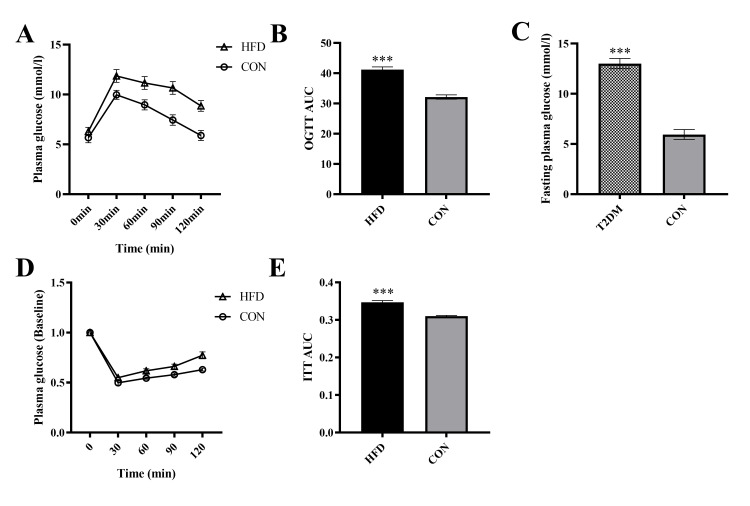
The effect of HFD on glucose metabolism in mice. A. OGTT, B. OGTT AUC,  C. FPG,  D. ITT,  E. ITT AUC.  *** p<0.001 (Compared to the CON group) OGTT:  oral glucose tolerance test; AUC: area under the curve; fasting plasma glucose; ITT: insulin tolerance test

The OGTT results showed that after 4 weeks of a high-fat diet, the AUC in the HFD group was significantly higher than in the CON group (t=38.58, p<0.001), indicating that the glucose regulation ability of the HFD group mice was impaired. The ITT results showed that the AUC in the HFD group was significantly higher than in the CON group (t=34.01, p<0.001), suggesting that the insulin sensitivity of the HFD group mice decreased, indicating that the HFD-fed mice developed insulin resistance (Figure 1).

Evaluation of the Experimental Periodontitis Mouse Model

**Figure 2:**
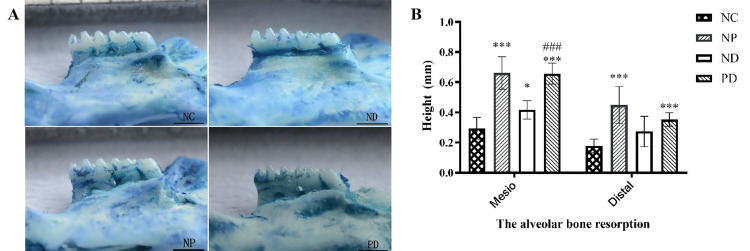
Silk ligature induces alveolar bone resorption in the right mandibular molars of mice. A. Staining of the mouse mandible with 0.5% methylene blue solution. B. The distance between the cemento-enamel junction and the alveolar crest on the mesial and distal sides of the right mandibular first molar. * p<0.05，*** p<0.001 (Compared to the NC group). ### p<0.001 (Compared to the ND group).

In the modeling of experimental periodontitis in mice, the gums showed significant redness and swelling, and some mice had tooth displacement. 0.5% methylene blue staining showed alveolar bone resorption at the mesial (F=41.902, p<0.001) and distal (F=14.424, p<0.001) sites of the right mandibular first molar in the mice (Figure 2). Pairwise comparisons of the distance from the cementoenamel junction to the alveolar crest at the mesial and distal sites of the right mandibular first molar showed that the distances in the ND, NP, and PD groups were greater than those in the NC group, with statistically significant differences (p<0.05). The distances in the NP and PD groups were greater than those in the ND group, also with statistically significant differences (p<0.05) (Figure 2).

Effects of Experimental Periodontitis on Body Weight and Glucose Metabolism in T2DM Mice

**Figure 3:**
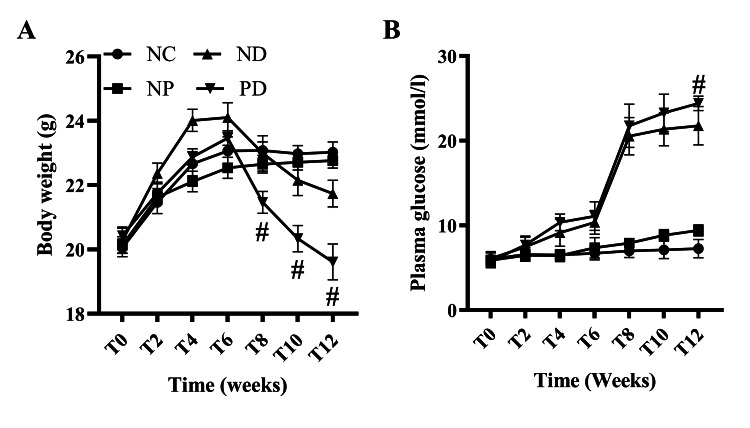
The effect of experimental periodontitis on the body weight and blood glucose levels of T2DM mice. A. Body weight, B. Blood glucose. # p<0.05 (Compared to the ND group).

Six weeks ago, the body weight of mice in each group increased significantly over time, with statistically significant differences (F=29.768, p<0.001). There were also significant differences in body weight between groups at various time points (F=3.554, p <0.05). There was a significant interaction effect between experimental periodontitis and time on the body weight of T2DM mice (F=2.713, p<0.01), indicating intergroup differences in body weight changes over time. Further analysis of the simple effect of experimental periodontitis on body weight in T2DM mice showed that the body weight of the PD group was significantly lower than that of the ND group starting from week 8 (p<0.05) (Figure 3).

FPG tests after 6 weeks of experimental periodontitis modeling showed that FPG was significantly higher in the NP, ND, and PD groups compared to the NC group (F=231.257, p<0.001). Further analysis of the simple effect of experimental periodontitis on blood glucose in T2DM mice showed that FPG in the PD group was higher than in the ND group, with statistically significant differences at week 12 (p<0.05) (Figure 3).

**Figure 4:**
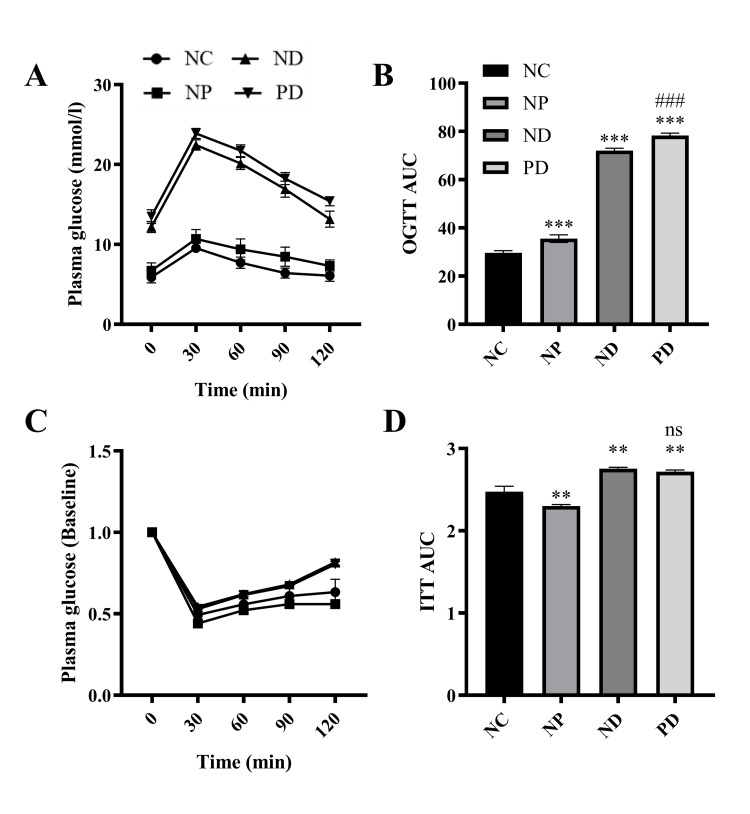
The effect of experimental periodontitis on glucose metabolism in T2DM mice. A.OGTT, B. OGTT AUC, C. ITT, D. ITT AUC. ** p<0.01, *** p<0.001(Compared to the NC group), ns p>0.05. ### p<0.001 (Compared to the ND group). OGTT:  oral glucose tolerance test; AUC: area under the curve; fasting plasma glucose; ITT: insulin tolerance test

At the end of week 12, OGTT and ITT results showed significant differences in oral glucose tolerance levels among NC, NP, ND, and PD groups of mice, with statistical significance (F=442.245, p<0.001). Pairwise comparison results showed that the oral glucose tolerance levels in the NP, ND, and PD groups were lower than those in the NC group (p<0.01); the oral glucose tolerance levels in the ND and PD groups were lower than those in the NP group (p<0.01); and the oral glucose tolerance level in the PD group was lower than that in the ND group (p<0.01). There were significant differences in insulin sensitivity among the NC, NP, ND, and PD groups of mice, with statistical significance (F=78.977, p<0.001). Insulin sensitivity levels in the ND and PD groups were lower than those in the NC group (p<0.01) and the NP group (p<0.01). The differences in insulin sensitivity levels among the remaining groups were not statistically significant(Figure 4).

Changes in Serum IL-17 in T2DM Mice with Experimental Periodontitis

ELISA results showed significant differences in serum IL-17 levels among NC, NP, ND, and PD groups of mice (H=25.641, p<0.001). Pairwise comparisons indicated that serum IL-17 levels in the ND group (9.95 ± 3.37) and PD group (5.45 ± 2.42) were lower than in the NC group (28.20 ± 5.88), with statistically significant differences (p<0.01). Serum IL-17 levels in the PD group (5.45 ± 2.42) were lower than in the NP group (27.88 ± 11.51), with statistically significant differences (p<0.01). There were no statistically significant differences between the other groups.

HE Staining of Mouse Small Intestine Tissue

**Figure 5:**
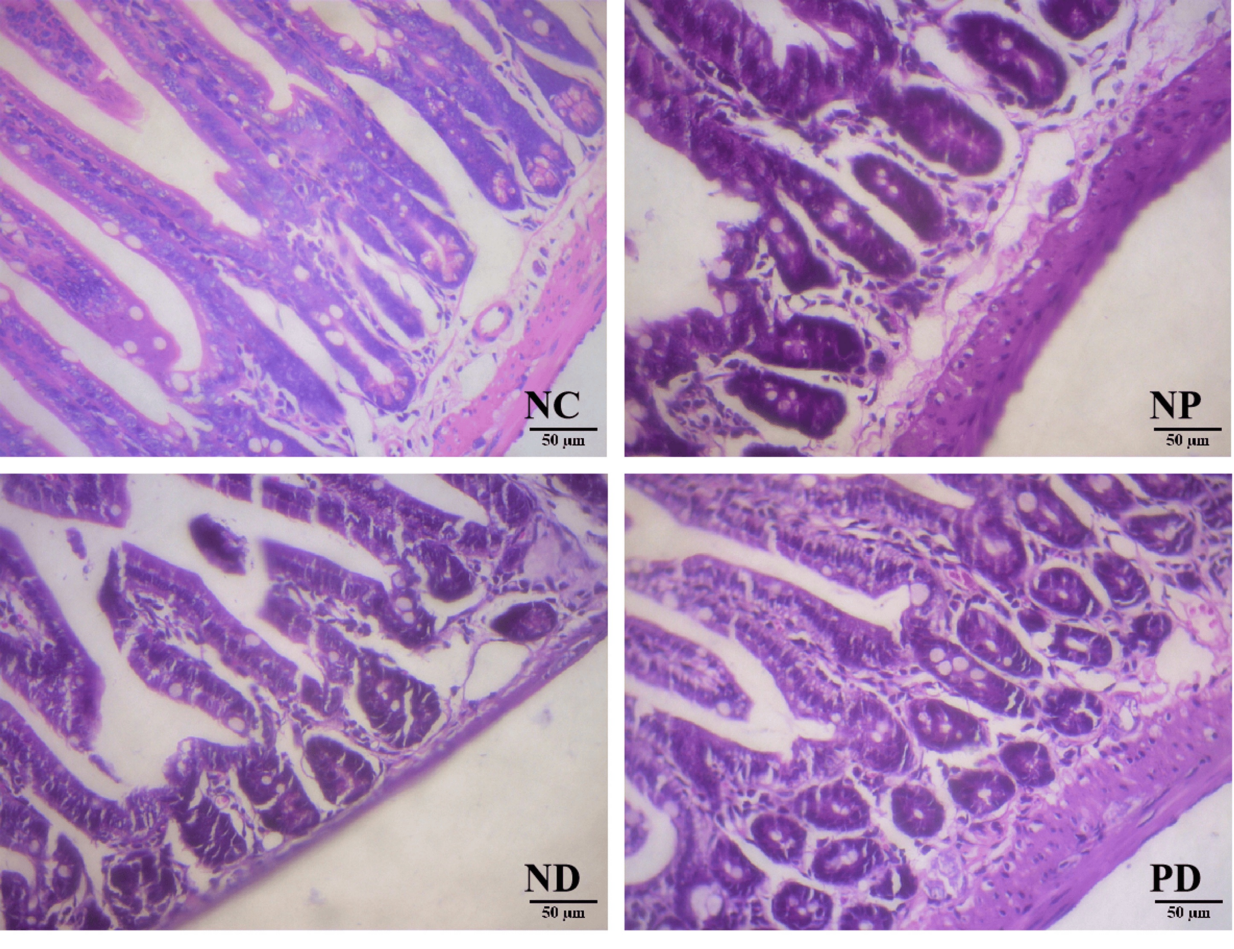
Small intestine HE staining (HE, X200).

HE staining results: The NC group showed normal villi and intestinal glands in the small intestine; the ND group had partially atrophied villi, increased mononuclear cells in the lamina propria, inflammatory cell infiltration in the lamina propria, vacuolar degeneration of intestinal epithelium, partial crypt hyperplasia, and reduced Paneth cells. The NP group was similar to the ND group but with significantly increased Paneth cells. The PD group showed no Paneth cells, partial crypt hyperplasia, increased apoptotic bodies in the crypts, and increased intraepithelial lymphocytes (Figure 5).

Alpha Diversity Analysis of Gut Microbiota

**Figure 6:**
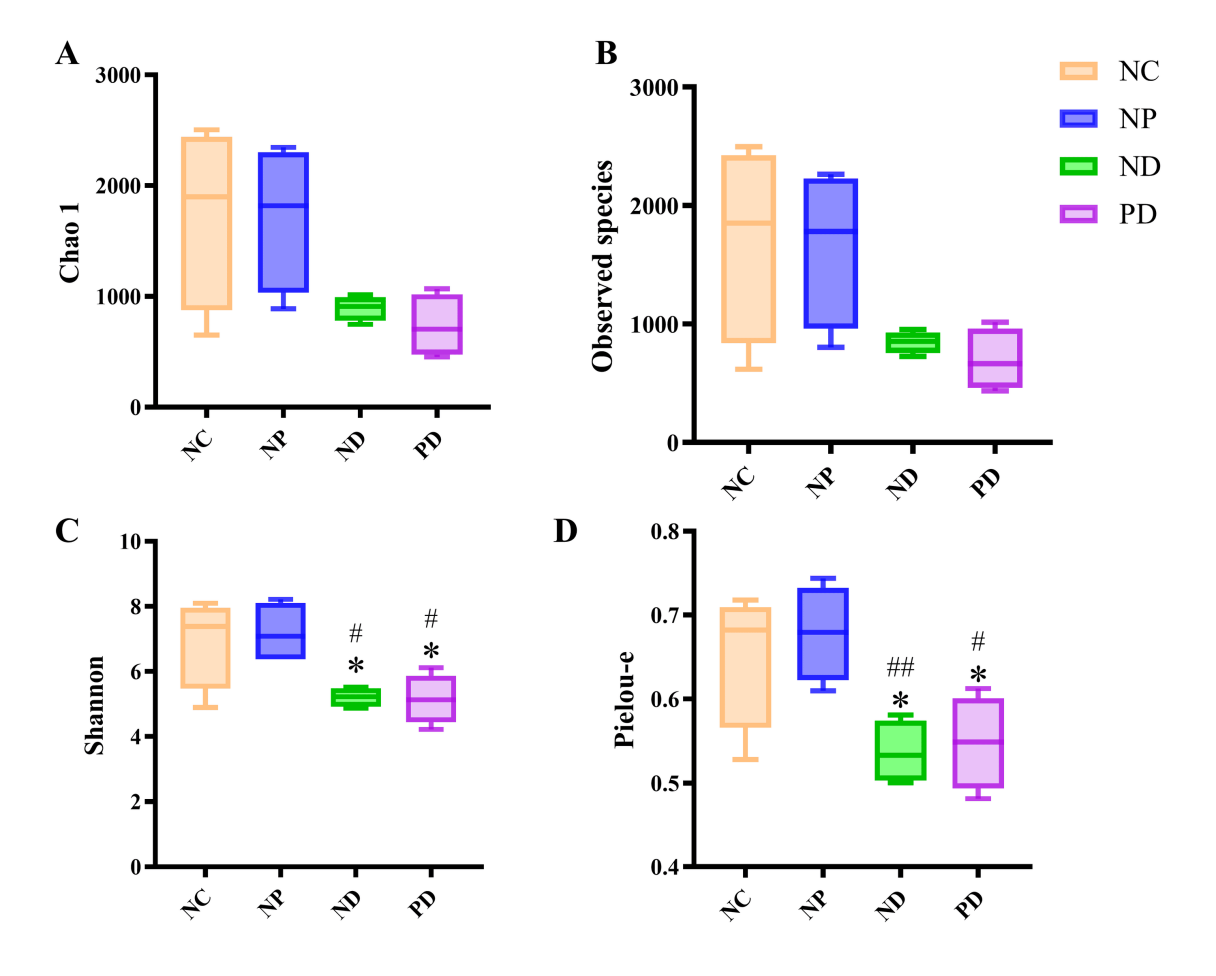
Analysis of gut microbiota α-diversity in mice with T2DM and experimental periodontitis A.The Chao1 index represents species richness, where a higher index indicates a greater number of species within the community. B. The Observed species index represents species richness, with a higher index reflecting a greater number of species within the community. C. The Shannon index represents species diversity, where a higher index indicates greater diversity. D. The Pielou-e index represents species evenness, with a higher value indicating greater evenness.* p<0.05 (compared to the NC group). # p<0.05, ## p<0.01 (compared to the NP group).

Analysis of the gut microbiota in mice showed no statistically significant differences in the Observed species index and Chao 1 index among the groups. However, the Shannon index and Pielou-e index of the gut microbiota in the ND group were significantly lower than those in the NC and NP groups, with statistically significant differences (p<0.05). Similarly, the Shannon index and Pielou-e index in the PD group were significantly lower than those in the NC and NP groups, with statistically significant differences (p<0.05) (Figure 6).

Gut Microbiota Composition Analysis

**Figure 7:**
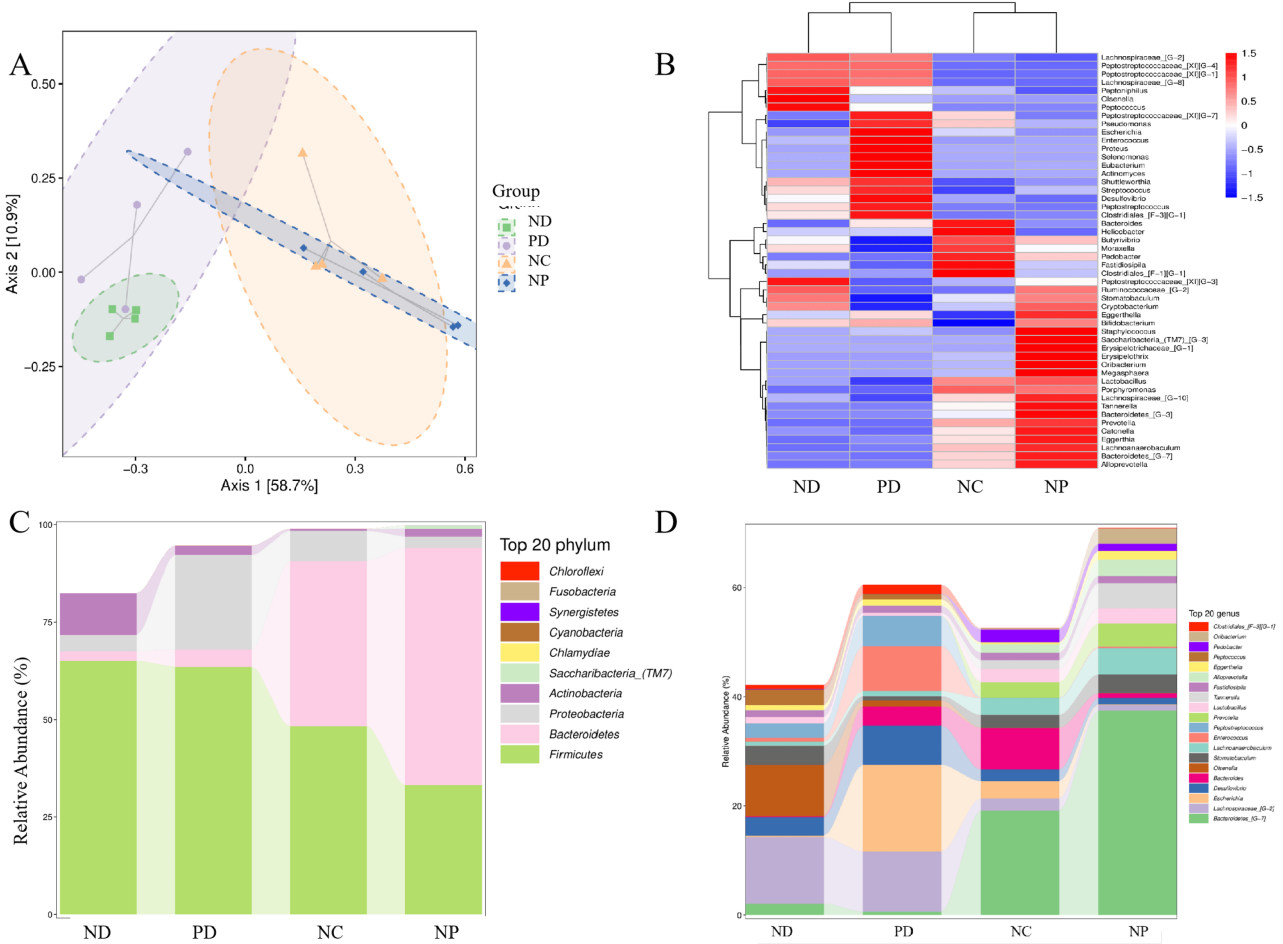
Analysis of gut microbiota composition in mice with T2DM and experimental periodontitis A. Principal Coordinates Analysis (PCoA), B. Species Clustering Heatmap, C. Relative Abundance Changes of Microbiota at the Phylum Level, D. Relative Abundance Changes of Microbiota at the Genus Level.

Species composition analysis and PCoA analysis of gut microbiota at the ASV/OTU level showed significant differences in gut microbiota composition between the NC group and the NP, ND, and PD groups, indicating that experimental periodontitis and diabetes altered the gut microbiota structure in mice to some extent. At the phylum level, Firmicutes in the gut of ND and PD groups were increased compared to the NC and NP groups (p<0.05); Bacteroidetes in the ND and PD groups were decreased compared to the NC and NP groups (p<0.05); Proteobacteria in the PD group were increased compared to the ND group (p<0.05); Saccharibacteria (TM7) were detected at higher levels in the NP group compared to the NC, ND, and PD groups (p<0.05) (Figure 7). At the genus level, the relative abundance of Oribacterium, Alloprevotella, Tannerella, Prevotella, Lachnoanaerobaculum, and Bacteroidetes_[G-7] was higher in the NP group compared to the NC group (p<0.05); the relative abundance of Bacteroidetes_[G-7] was lower in the ND and PD groups compared to the NC group (p<0.05); the relative abundance of Lachnospiraceae_[G-2] was higher in the ND and PD groups compared to the NC group (p<0.05); the relative abundance of Olsenella was lower in the PD group compared to the ND group (p<0.05); the relative abundance of Escherichia, Desulfovibrio, Bacteroides, Enterococcus, and Peptostreptococcus was higher in the PD group compared to the ND group (p<0.05) (Figure 7).

Species Difference Analysis and Marker Species

**Figure 8:**
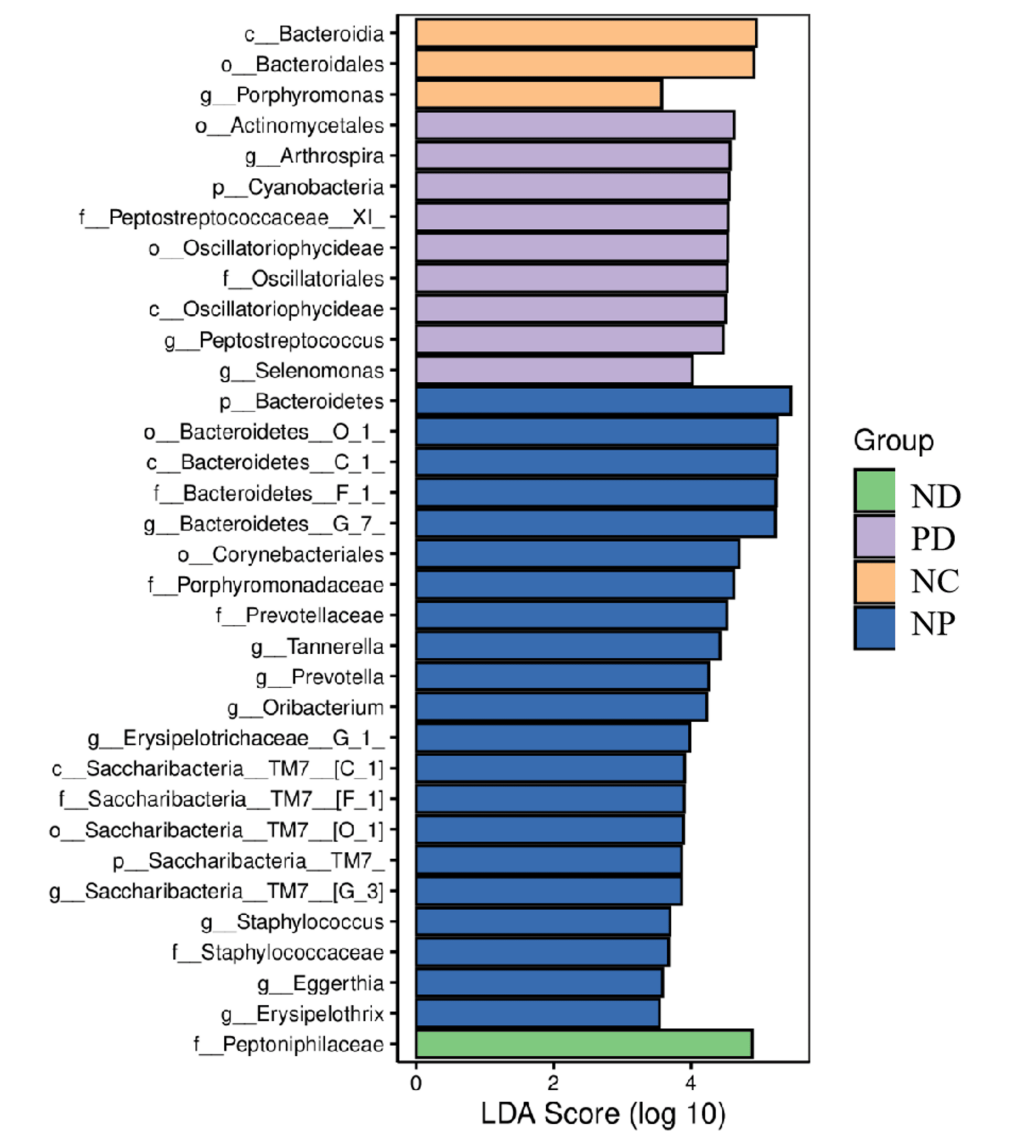
LEfSe bar chart of indicator species. LEfSe: linear discriminant analysis effect size

LEfSe analysis results showed that there were corresponding dominant bacteria in the gut microbiota of each group of mice (LDA value > 3) (Figure 8).

Correlation Analysis of Changes in Gut Microbiota at the Phylum and Genus Levels with Serum IL-17 Levels and Blood Glucose

Spearman correlation analysis was performed between the phyla with statistically significant differences in gut microbiota and serum IL-17 levels in each group of mice: The abundance of Proteobacteria (9.8 ± 13.09)% was moderately negatively correlated with serum IL-17 levels (20.13 ± 15.97) (p<0.05); the abundance of Bacteroidetes (27.59 ± 28.56)% and the Bacteroidetes / Firmicutes ratio (1.03 ± 1.76) were moderately positively correlated with serum IL-17 levels (20.13 ± 15.97) (p<0.01). Spearman correlation analysis was performed between the phyla with statistically significant differences in gut microbiota and FPG before euthanasia in each group of mice: The abundance of Bacteroidetes (27.59 ± 28.56)% and the Bacteroidetes / Firmicutes ratio (1.03 ± 1.76) were moderately negatively correlated with FPG (15.74 ± 7.76) (p<0.01). Spearman correlation analysis was also performed between serum IL-17 levels and FPG before euthanasia in each group of mice: Serum IL-17 levels (20.13 ± 15.97) were strongly negatively correlated with FPG (15.74 ± 7.76) (p<0.01) (Table 2).

**Table 2:** Distance matrix of bacteria at the phylum level in relation to IL-17 and blood glucose. The blood glucose values included in this analysis are the values measured before the mice were sacrificed. * p< 0.05 , ** p< 0.01. FPG: fasting plasma glucose; IL-17: interleukin 17

	Actinobacteri	Bacteroidetes	Firmicutes	Bacteroidetes/Firmicutes	Proteobacteria	IL-17
Firmicutes	0.485	-0.597^*^				
Bacteroidetes/Firmicutes	-0.441	0.988^**^	-0.650^**^			
Proteobacteria	-0.009	-0.371	0.144	-0.35		
IL-17	-0.375	0.696^**^	-0.422	0.686^**^	-0.606^*^	
FPG	0.548^*^	-0.710^**^	0.448	-0.676^**^	0.392	-0.846^**^

Spearman correlation analysis was performed between the genera with statistically significant differences in gut microbiota and serum IL-17 levels, and FPG before euthanasia in each group of mice: the genera Prevotella, Alloprevotella, unclassified_Prevotellaceae, Tannerella, Bacteroidetes_[G-7], Porphyromonas, Erysipelothrix, Megasphaera, unclassified_Porphyromonadaceae, and unclassified_Bacteroidetes were moderately positively correlated with serum IL-17 levels and moderately negatively correlated with FPG. The genera Arthrospira, Selenomonas, Peptococcus, and Actinomyces were moderately negatively correlated with serum IL-17 levels and moderately positively correlated with FPG. The genera unclassified_Coriobacteriia and Peptostreptococcus were strongly negatively correlated with serum IL-17 levels and moderately positively correlated with FPG (p<0.01) (Table 3).

**Table 3:** Distance matrix of bacteria at the genus level in relation to IL-17 and blood glucose. The blood glucose values included in this analysis are the fasting plasma glucose (FPG) values measured before the mice were sacrificed. * p< 0.05 , ** p< 0.01.

	IL-17	FPG
g__Arthrospira	-.537^*^	.711^**^
g__Selenomonas	-.548^*^	.725^**^
g__Prevotella	.591^*^	-.530^*^
g__Oribacterium	.637^**^	-0.444
g__Peptococcus	-.671^**^	.704^**^
g__Alloprevotella	.683^**^	-.672^**^
g__unclassified_Prevotellaceae	.698^**^	-.658^**^
g__Actinomyces	-.700^**^	.735^**^
g__Tannerella	.732^**^	-.743^**^
g__unclassified_Bacteroidetes	.738^**^	-.630^**^
g__Bacteroidetes_[G-7]	.751^**^	-.658^**^
g__Porphyromonas	.769^**^	-.819^**^
g__Erysipelothrix	.774^**^	-.749^**^
g__Megasphaera	.778^**^	-.596^*^
g__unclassified_Porphyromonadaceae	.795^**^	-.755^**^
g__unclassified_Coriobacteriia	-.806^**^	.796^**^
g__Peptostreptococcus	-.810^**^	.647^**^

## Discussion

The relationship between periodontitis and systemic diseases such as cardiovascular disease, respiratory disease, diabetes, Alzheimer's disease, cancer, adverse pregnancy outcomes, and multiple myeloma is gradually being confirmed. Research indicates a bidirectional relationship between periodontitis and diabetes, high glucose environments exacerbate periodontal tissue destruction in patients and experimental animals with periodontitis, while periodontitis, in turn, affects glycemic control and systemic inflammation levels in diabetic patients [10]. Various microorganisms, host immune responses, and environmental factors play a synergistic role in the progression of chronic inflammatory diseases [3]. With the development of microbial ecology, the role of gut microbiota dysbiosis in metabolic diseases is gaining increasing attention

Animal models are important tools for studying the pathogenesis of periodontitis, and the silk ligature-induced periodontitis model in experimental animals has been widely used to study the etiology and mechanisms of periodontitis and its association with systemic diseases. The silk ligature, serving as a bacterial carrier for experimental periodontitis, creates an environment conducive to plaque accumulation. The bone loss induced by the silk ligature is comparable to that induced by the combination of ligature and local injection of *Porphyromonas gingivalis* [13], while avoiding the impact of local lipopolysaccharide or *Porphyromonas gingivalis* injection on the gut microbiota. The clinical manifestations of periodontitis include periodontal tissue inflammation and attachment loss. In this experiment, significant alveolar bone resorption was observed in the NP and PD groups, indicating that the silk ligature successfully induced experimental periodontitis, consistent with previous studies [14].

Silk ligature-induced periodontal inflammation exacerbated weight loss and impaired oral glucose tolerance in T2DM mice, possibly due to the comorbidity of experimental periodontitis and T2DM leading to decreased tolerance and increased nutritional consumption in the mice. Oral glucose tolerance test and insulin sensitivity detection are important reference indicators for the diagnosis and prognosis of T2DM patients. Experimental periodontitis may aggravate glucose tolerance impairment in T2DM mice. Insulin sensitivity in T2DM mice with experimental periodontitis was slightly reduced but not statistically significant. This might be related to the selective destruction of some islet cells by STZ, leading to a compensatory increase in insulin sensitivity in these mice. Studies have shown that diabetes affects bone formation in periodontal tissues, with high levels of glucose, reactive oxygen species, and advanced glycation end products found in the periodontal tissues of diabetic patients. This leads to increased activation of NF-κB and elevated expression of inflammatory cytokines (such as TNF and IL-1).In this experiment, compared to the NC group, the ND group mice exhibited alveolar bone loss. Studies indicate that animals like mice generally do not spontaneously develop periodontitis. This may be due to the high glucose environment increasing osteoclast activity and alveolar bone resorption [15].

The small intestine plays a crucial role in metabolic homeostasis, both physiologically and pathophysiologically [16]. IL-17 promotes the integrity of intestinal epithelial cells by regulating tight junction proteins, thereby preventing the entry of luminal contents and commensal organisms.IL-17 has multiple cellular sources, in the small intestine, Paneth cells are an important source of IL-17, in addition to the commonly recognized CD4+ T cells [17]. In mice, the absence of IL-17R in Paneth cells leads to an increased inflammatory transcription profile in the ileum and heightened severity of experimentally induced ileitis, which is associated with reduced gut microbiota diversity [18]. Studies have shown that treating diabetic mice with the insulin receptor antagonist (S961) results in impaired gut barrier epithelial function, characterized by increased epithelial permeability and disrupted cellular junction integrity. Early severe gut dysbiosis is also observed, marked by the proliferation of pro-inflammatory Proteobacteria and the failure of Paneth cell antimicrobial defenses [19]. In this study, increased intraepithelial lymphocytes, reduced Paneth cells, and increased apoptotic bodies in the crypts were observed in the small intestine tissue of the PD group, suggesting that experimental periodontitis may exacerbate intestinal mucosal damage in T2DM mice. The trend in IL-17 changes may be related to the presence or absence of Paneth cells in the small intestine, but quantitative analysis of IL-17+ Paneth cells is needed for verification. Additionally, in this study, T2DM mice were induced by feeding them a high-fat diet (HFD) combined with intraperitoneal injection of STZ. Given the cytotoxic effects of STZ, the increase in chronic inflammation-related cells in the small intestinal mucosa of the ND and PD groups may also be related to the T2DM modeling method.

The gut microbiota regulates intestinal immunity and barrier functions, which are crucial for maintaining the body's internal balance. The homeostasis of the gut microbiota is influenced by various factors, including diet, antibiotics, other medications, and bacterial and viral infections. Studies have shown that *Porphyromonas gingivalis *induces gut microbiota dysbiosis by disrupting metabolic and immune pathways, thereby promoting the progression of non-alcoholic fatty liver disease [20]. Periodontitis may influence diabetes by inducing gut microbiota dysbiosis through the influx of salivary microorganisms [21]. In this study, the gut microbiota diversity and evenness were reduced in both T2DM mice and T2DM mice with experimental periodontitis. However, there was no difference in richness, diversity, and evenness between the two groups, possibly due to T2DM dominating the gut microbiota in this model. Over 50 known phyla of gut microorganisms exist, predominantly anaerobes. Firmicutes and Bacteroidetes are the primary bacterial phyla residing in the intestines of healthy humans and mice, accounting for about 90% and playing a crucial role in maintaining gut homeostasis [22]. In contrast, Proteobacteria are less common, such as Escherichia and Enterobacteriaceae [23]. The ratio of Firmicutes, Bacteroidetes, and the Bacteroidetes/Firmicutes ratio, along with Proteobacteria, are significant indicators of gut homeostasis. In neonatal mice, Proteobacteria is the dominant phylum, and Proteobacteria-specific IgA maintains relative bacterial population balance and controls microbiota maturation, after which Proteobacteria are suppressed. Abnormal growth of Proteobacteria can lead to energy imbalances between different species and inhibit the growth of other species, thereby inducing disease [21]. Therefore, experimental periodontitis may increase the abundance of Proteobacteria in the gut microbiota of T2DM mice, thereby affecting glycemic control. At the genus level, Escherichia, Desulfovibrio, Bacteroides, Enterococcus, and Peptostreptococcus may be the dominant genera in mice with comorbid experimental periodontitis and T2DM.

The gastrointestinal tract has highly complex mechanisms for environmental homeostasis regulation, and the gut microbiota may mediate the impact of periodontitis on diabetes [24]. IL-17 has a protective role in the intestinal barrier. In the early stages of metabolic diseases, serum IL-17/IL-22 levels decrease along with changes in the gut microbiome [25]. As a dual-function cytokine, IL-17 production can also be regulated by the gut microbiota [26]. In HFD-related metabolic diseases, IL-17 plays a protective role in the intestinal mucosa, and HFD-related microbiota dysbiosis leads to a reduction in lamina propria Th17 cells [27]. Additionally, HFD induces gut microbiota dysbiosis, impairing intestinal immune defense, including a reduction in IL-17-related T cells in the gut. These changes in gut immunity precede the onset of diabetes [28,29]. Serum IL-17 levels can partially indicate the ratio of Bacteroidetes, Bacteroidetes/Firmicutes, and the balance of Proteobacteria in the mouse gut. The gut microbiota and IL-17 in T2DM mice with experimental periodontitis are related to their glucose metabolism. However, the specific mechanisms of their regulatory relationship with the gut microbiota require further research to better understand the impact of periodontitis on glycemic control, complications, prediabetes, and the incidence of new diabetes cases.

Most previous studies have focused on the relationship between periodontitis and diabetes. The novelty of this study lies in the innovative combination of a T2DM and experimental periodontitis comorbidity model. IL-17 plays a protective role in the intestinal barrier by regulating tight junction proteins to maintain the integrity of intestinal epithelial cells, thereby preventing luminal contents and symbiotic organisms from entering. This study is the first to investigate the correlation between the composition of the gut microbiota, serum IL-17 levels, and glucose metabolism in mice with both periodontitis and T2DM. It explores the impact of periodontopathogenic bacteria on the gut microbiota of diabetic mice and examines the effects of gut microbiota dysbiosis on glucose metabolism regulation under the induction of periodontitis. This research is significant for advancing the understanding of the "oral-gut-immune axis" mechanism by which periodontitis affects glucose metabolism in T2DM mice.

This study is limited to an experimental animal model, and further in vitro experiments are needed to validate the related mechanisms. The pathogenesis of T2DM is complex, and the mice in this study had a relatively uniform diet, limiting the interpretation of observed phenomena to this specific model. Additional T2DM mouse models and clinical studies are required to confirm these findings. The gut microbiota and gut barrier immunity play significant roles in the comorbidity of periodontitis and diabetes. Future research will focus on the specific regulatory mechanisms of the gut microbiota and IL-17 in the context of periodontitis and T2DM comorbidity, providing theoretical support for the role of the "oral-gut-immune axis" in glucose metabolism regulation.

## Conclusions

This study investigated the compositional characteristics of gut microbiota in mice with type 2 diabetes mellitus (T2DM) and experimental periodontitis, as well as their correlation with serum IL-17 levels. The findings revealed that both experimental periodontitis and diabetes partially altered the gut microbiota structure in mice. The comorbidity of experimental periodontitis and T2DM may exacerbate glucose metabolism impairment in T2DM mice by increasing the abundance of Proteobacteria and causing small intestinal mucosal damage. Serum IL-17 levels may serve as an indicator of the balance of Bacteroidetes, the Bacteroidetes/Firmicutes ratio, and Proteobacteria in the gut of mice. This suggests that serum IL-17 levels could be a potential marker for gut dysbiosis in T2DM mice with experimental periodontitis.
